# Association between peripheral blood T cell subsets and clinical disability in multiple sclerosis patients

**DOI:** 10.3389/fneur.2026.1843351

**Published:** 2026-07-20

**Authors:** Penju Liu, Wei Liu, Xiaoting Zhang, Peng Yuan

**Affiliations:** 1 Department of Neurology, Beijing Anzhen Hospital, Capital Medical University, Beijing, China; 2 Department of Neurology, The Second Hospital & Clinical Medical School, Lanzhou University, Lanzhou, Gansu Province, China; 3 Department of Neurology, Hospital of University of Jinan, Jinan, Shandong Province, China

**Keywords:** CD4+/CD8+ ratio, disability assessment, multiple sclerosis, neuroimmunology, T cell subsets

## Abstract

**Background:**

Multiple sclerosis (MS) is a chronic immune-mediated disorder involving complex interactions among adaptive and innate immune pathways. The relationship between peripheral blood T cell subsets and clinical disability remains inadequately characterized. This study investigated associations between T cell subsets (CD4+, CD8+, CD4+/CD8 + ratio) and Expanded Disability Status Scale (EDSS) scores in MS patients.

**Methods:**

We conducted a cross-sectional study of 48 MS patients who underwent EDSS scoring and flow cytometric analysis of peripheral blood T cell subsets. Clinical and treatment-related characteristics were also collected. Correlations between T cell parameters and EDSS scores were analyzed using Spearman correlation and multivariable linear regression. Exploratory sensitivity analyses were performed to evaluate the potential influence of disease-modifying therapy (DMT) exposure on the association between CD4+/CD8 + ratio and EDSS.

**Results:**

Forty-eight MS patients (mean age 35.1 ± 5.8 years, 72.9% female) with mean EDSS score of 3.20 ± 1.50 were included. Mean CD4+/CD8 + ratio was 2.76 ± 1.08 and correlated negatively with EDSS scores (*r* = −0.345, *p* = 0.016). Stratified analysis showed progressive decline across disability groups: mild (EDSS 0–2.5): 3.19 ± 1.37; moderate (EDSS 3.0–4.5): 2.64 ± 0.81; severe (EDSS ≥ 5.0): 2.13 ± 0.56 (*p* = 0.050). In the selected covariate-adjusted multivariable model, the association between CD4+/CD8 + ratio and EDSS was attenuated (*β* = −0.350, *p* = 0.084). Exploratory treatment-related sensitivity analyses further suggested that this association may be influenced by DMT exposure.

**Conclusion:**

CD4+/CD8 + ratio was negatively associated with clinical disability in this cross-sectional MS cohort, and stratified analysis showed a progressive decline across disability severity groups. These findings suggest that CD4+/CD8 + ratio may represent an exploratory peripheral immune indicator associated with disability status in MS.

## 1 Introduction

Multiple sclerosis (MS) is a chronic inflammatory and neurodegenerative disorder of the central nervous system (CNS), affecting approximately 2.8 million people worldwide (1). The disease is characterized by focal demyelinating lesions, axonal damage, and progressive disability (2). Although T cells are central contributors to MS immunopathology, MS is now recognized as a complex immune-mediated disorder involving coordinated interactions among CD4 + and CD8 + T cells, B cells, innate immune cells, and compartmentalized inflammatory processes within the CNS (3–5). B cells contribute to antigen presentation, cytokine production, antibody-mediated responses, and meningeal inflammatory aggregates, findings that are also supported by the clinical efficacy of B cell-depleting strategies in MS (4, 5). While the precise etiology remains incompletely understood, converging evidence implicates dysregulated adaptive and innate immune responses as central pathogenic mechanisms in disease initiation and progression (3–5).

The adaptive immune system, particularly T lymphocytes, plays a critical role in MS pathogenesis. CD4 + T helper cells, especially pro-inflammatory Th1 and Th17 subsets, have been implicated in initiating and perpetuating CNS inflammation (5, 6). These cells produce interferon-gamma (IFN-*γ*) and interleukin-17 (IL-17), cytokines that promote blood–brain barrier disruption, microglial activation, and oligodendrocyte damage (7). CD8 + cytotoxic T cells represent another crucial component of MS immunopathology. These cells are abundant in active MS lesions and can directly induce oligodendrocyte and neuronal damage through perforin and granzyme-mediated cytotoxicity (8, 9). In addition, recent studies have highlighted broader immune dysregulation in MS, including altered activation states and functional imbalance across circulating T-cell subsets, suggesting that peripheral immune signatures may capture part of the immunopathological context of the disease (10–13).

Recent studies have highlighted dysregulated CD4+/CD8 + T cell ratios in MS patients, though the clinical significance of these alterations remains debated (10, 11). The balance between different T cell subsets may reflect the overall inflammatory status and disease activity. Some investigations have suggested that peripheral T cell subset imbalances correlate with disease progression and treatment responses (12, 13). However, comprehensive analyses correlating peripheral blood T cell profiles with standardized disability measures are limited.

Disease monitoring in MS relies heavily on clinical assessment using the Expanded Disability Status Scale (EDSS), which provides standardized disability measurement ranging from 0 (normal neurological examination) to 10 (death due to MS) (14). However, EDSS has recognized limitations including non-linearity, emphasis on ambulatory function, and insensitivity to cognitive impairment (15). Current clinical practice lacks readily accessible biomarkers that objectively correlate with disability status and could complement clinical assessment.

Understanding the relationship between peripheral T cell profiles and clinical disability status could provide insights into MS pathophysiology and identify potential peripheral immunological biomarkers for disease monitoring. Peripheral blood sampling offers practical advantages over cerebrospinal fluid analysis or tissue biopsies, making T cell profiling an attractive approach for routine clinical application (16). If peripheral T cell subset distributions are associated with disability status, they may provide an objective immunological correlate to complement EDSS assessment and support future biomarker-oriented research.

Therefore, we conducted this study with the following objectives: (1) to comprehensively characterize peripheral blood T cell subset distributions in MS patients; (2) to investigate associations between T cell subset profiles (CD4+, CD8+, and CD4+/CD8 + ratio) and EDSS scores as a measure of clinical disability; and (3) to identify T cell subset patterns that may serve as potential biomarkers associated with disability status. The specific novelty of this study lies in integrating peripheral blood flow cytometric T-cell profiling with EDSS-based disability stratification and multivariable analysis to evaluate the clinical interpretability of CD4+/CD8 + ratio as a readily measurable peripheral immune indicator in MS.

## 2 Materials and methods

### 2.1 Study design and participants

This cross-sectional observational study was conducted at a tertiary neurology center between January 2023 and December 2024. MS patients fulfilling the 2017 revised McDonald diagnostic criteria (17) were consecutively recruited from the outpatient clinic. Inclusion criteria were: (1) confirmed MS diagnosis according to the 2017 McDonald criteria; (2) age 18–65 years; (3) clinically stable disease (no clinical relapse or corticosteroid treatment within 30 days prior to enrollment); (4) voluntary provision of written informed consent. Exclusion criteria included: (1) other autoimmune or inflammatory diseases; (2) active infection within 4 weeks; (3) immunosuppressive therapy within 3 months (except for first-line disease-modifying therapies); (4) pregnancy or breastfeeding; and (5) other significant neurological conditions.

This study was approved by the Institutional Ethics Committee of Beijing Anzhen Hospital, Capital Medical University. All procedures were conducted in accordance with the Declaration of Helsinki (2013 revision) and Good Clinical Practice guidelines. All participants provided written informed consent after receiving detailed information about the study objectives, procedures, risks, and benefits. Participants retained the right to withdraw at any time without consequences to their clinical care. Patient confidentiality was strictly maintained, with all data anonymized and securely stored.

### 2.2 Clinical assessment

Neurological disability was assessed using the Expanded Disability Status Scale (EDSS) by trained neurologists. The EDSS is a standardized 20-point scale ranging from 0 (normal neurological examination) to 10 (death due to MS), with increments of 0.5 points. Scores from 0 to 3.5 primarily reflect impairment in functional systems (pyramidal, cerebellar, brainstem, sensory, bowel/bladder, visual, cerebral, and other functions) with preserved ambulatory capacity. Scores from 4.0 to 7.5 increasingly reflect ambulatory limitations. Higher EDSS scores indicate greater neurological disability and functional impairment.

Disease course was classified based on clinical and radiological features as relapsing–remitting MS (RRMS, characterized by distinct relapses with full or partial recovery and no disease progression between relapses), secondary progressive MS (SPMS, defined by initial relapsing–remitting course followed by progressive worsening with or without occasional relapses), primary progressive MS (PPMS, marked by continuous disease progression from onset without distinct relapses), or clinically isolated syndrome (CIS, a first episode of neurological symptoms lasting at least 24 h with features suggestive of MS but not yet fulfilling MS diagnostic criteria) according to established criteria (18). Disease duration was calculated from the time of first symptom onset. Demographic data and treatment exposure were recorded, including current DMT status, DMT type, treatment duration, and prior treatment history. Current DMT status was defined according to the recorded current DMT agent at enrollment. Patients without a recorded current DMT agent were classified as untreated and were further categorized as treatment-naïve or previously treated but discontinued according to prior treatment history. Treatment exposure was considered when interpreting the immunological findings because selected DMTs may have marked effects on peripheral lymphocyte subsets. Because fingolimod and anti-CD20 therapy may exert particularly strong effects on circulating lymphocyte subsets, patients receiving fingolimod or ofatumumab were additionally considered as a strong lymphocyte-modifying DMT subgroup in exploratory sensitivity analyses.

### 2.3 Blood sample collection and processing

Peripheral venous blood (10 mL) was collected in EDTA tubes on the same day as clinical assessment. Samples were processed within 4 h of collection. Peripheral blood mononuclear cells (PBMCs) were isolated using Ficoll-Paque density gradient centrifugation according to standard protocols. Complete blood counts were performed to obtain absolute lymphocyte counts and neutrophil-to-lymphocyte ratios (NLR).

### 2.4 Flow cytometric analysis

PBMCs were stained for surface markers using fluorochrome-conjugated antibodies: CD3-FITC, CD4-APC, CD8-PE (BD Biosciences). Flow cytometry was performed on a BD FACSCanto II instrument, acquiring at least 100,000 events per sample. Data were analyzed using FlowJo software version 10.8.

T cell subsets were identified using the following gating strategy: lymphocytes were gated based on forward and side scatter characteristics, followed by CD3 + gating for total T cells. CD4 + and CD8 + populations were identified from CD3 + cells. Results were expressed as percentages of parent populations. The CD4+/CD8 + ratio was calculated using absolute CD4 + and CD8 + T-cell counts. Absolute CD4 + and CD8 + counts were calculated based on lymphocyte counts and flow cytometry percentages.

### 2.5 Statistical analysis

Statistical analyses were performed using SPSS version 26.0 and GraphPad Prism version 9.0. Continuous variables were assessed for normality using the Shapiro–Wilk test (*p* > 0.05 indicating normal distribution) complemented by visual inspection of Q-Q plots and histograms and presented as mean ± standard deviation (SD) for normally distributed data or median (interquartile range, IQR) for non-normally distributed data. Categorical variables were expressed as frequencies and percentages.

Correlations between T cell subsets and EDSS scores were analyzed using Spearman’s rank correlation coefficient. The strength of correlation was interpreted as weak (*r* = 0.10–0.39), moderate (*r* = 0.40–0.69), or strong (*r* ≥ 0.70). Multivariable linear regression analysis was conducted to evaluate the association between immunological variables and EDSS scores. The final model included CD4+/CD8 + ratio, CD8 + T-cell percentage, and disease duration as independent variables. Treatment-related variables were not included in the primary multivariable model.

To address the potential confounding effect of DMT exposure, exploratory sensitivity analyses were additionally performed. These included models further incorporating current DMT status, strong lymphocyte-modifying DMT exposure defined as fingolimod or ofatumumab use, and DMT-type indicators. An additional sensitivity analysis was performed after excluding patients receiving fingolimod or ofatumumab.

Subgroup analyses were performed stratifying patients by EDSS severity: mild disability (EDSS 0–2.5), moderate disability (EDSS 3.0–4.5), and severe disability (EDSS ≥5.0). Between-group comparisons used one-way ANOVA for continuous variables. A two-tailed *p* value < 0.05 was considered statistically significant. Given the exploratory nature of the study and the limited sample size, no formal multiple-comparison correction was applied. Therefore, the statistical findings should be interpreted cautiously.

## 3 Results

### 3.1 Patient characteristics

Forty-eight MS patients were enrolled in the study. Demographic and clinical characteristics are summarized in Table 1. The cohort included 35 females (72.9%) and 13 males (27.1%) with a mean age of 35.1 ± 5.8 years (range: 21–46 years). Disease subtype distribution showed 26 patients (54.2%) with RRMS, 5 patients (10.4%) with SPMS, 7 patients (14.6%) with PPMS, and 10 patients (20.8%) classified as CIS or other subtypes. Mean disease duration was 28.8 ± 15.0 months (range: 6–66 months). The mean EDSS score was 3.20 ± 1.50 (range: 0.0–6.0), with a median value of 3.25. Thirty-five patients (72.9%) were receiving disease-modifying therapy at the time of enrollment, including teriflunomide in 23 patients, fingolimod in 7 patients, and ofatumumab in 5 patients. Mean treatment durations were 18.65 ± 6.17 months for teriflunomide, 12.57 ± 5.00 months for fingolimod, and 9.40 ± 2.70 months for ofatumumab. Among the remaining 13 patients, 9 were treatment-naïve and 4 had previously received treatment but had discontinued therapy before enrollment. Treatment information is summarized in Table 1 and Supplementary Table S1.

**Table 1:** Demographic and Clinical Characteristics of MS Patients (*n* = 48).

Characteristic	Value
Sex, n (%)	
Female	35 (72.9)
Male	13 (27.1)
Age (years)	
Mean ± SD	35.1 ± 5.8
Range	21–46
Disease subtype, n (%)	
RRMS	26 (54.2)
SPMS	5 (10.4)
PPMS	7 (14.6)
CIS/Others	10 (20.8)
Disease duration (months)	
Mean ± SD	28.8 ± 15.0
Range	6–66
EDSS score	
Mean ± SD	3.20 ± 1.50
Median (range)	3.25 (0.0–6.0)
DMT status, n (%)	
Currently receiving	35 (72.9)
Treatment-naïve or discontinued	13 (27.1)
Current DMT type, n (%)	
Teriflunomide	23 (47.9)
Fingolimod	7 (14.6)
Ofatumumab	5 (10.4)
Treatment duration, months	
Teriflunomide	18.65 ± 6.17
Fingolimod	12.57 ± 5.00
Ofatumumab	9.40 ± 2.70
Untreated subgroup, n	
Treatment-naïve	9
Previously treated but discontinued	4

Abbreviations: CIS, clinically isolated syndrome; DMT, disease-modifying therapy; EDSS, Expanded Disability Status Scale; PPMS, primary progressive multiple sclerosis; RRMS, relapsing-remitting multiple sclerosis; SD, standard deviation; SPMS, secondary progressive multiple sclerosis.

### 3.2 T cell subset profiles

Flow cytometric analysis revealed the following T cell subset distributions (Table 2). The mean percentage of CD4 + T cells was 45.3 ± 10.5% of total CD3 + T cells (range: 23.2–69.1%), while CD8 + T cells comprised 27.3 ± 7.3% (range: 11.9–45.2%). The mean CD4+/CD8 + ratio was 2.76 ± 1.08 (range: 1.21–6.58). This ratio was calculated from absolute CD4 + and CD8 + T-cell counts. Because no age- and sex-matched healthy control group was included, no formal comparison with healthy populations was made. The mean absolute CD4 + count was 760.7 ± 206.9 cells/μL (range: 375.0–1190.0), and the mean absolute CD8 + count was 310.7 ± 125.4 cells/μL (range: 57.0–691.0).

**Table 2:** Peripheral Blood T Cell Subset Profiles in MS Patients (*n* = 48).

Parameter	Mean ± SD (Range)
CD4+ T cells	
Percentage (%)	45.3 ± 10.5 (23.2–69.1)
Absolute count (cells/μL)	760.7 ± 206.9 (375–1190)
CD8+ T cells	
Percentage (%)	27.3 ± 7.3 (11.9–45.2)
Absolute count (cells/μL)	310.7 ± 125.4 (57–691)
CD4+/CD8+ ratio	2.76 ± 1.08 (1.21–6.58)
Other laboratory parameters	
WBC count (×10^9^/L)	6.69 ± 2.23 (1.42–13.44)
Neutrophil-to-lymphocyte ratio	3.64 ± 2.13 (0.28–8.92)

Abbreviations: SD, standard deviation; WBC, white blood cell.

### 3.3 Correlations between T cell subsets and EDSS scores

In unadjusted correlation analysis, a statistically significant negative association between CD4+/CD8 + ratio and EDSS scores (*r* = −0.345, *p* = 0.016), indicating that lower CD4+/CD8 + ratios were associated with greater clinical disability (Table 3). This represents a weak-to-moderate negative correlation, suggesting that as the CD4+/CD8 + ratio decreases, clinical disability as measured by EDSS tends to increase.

**Table 3:** Correlations Between T Cell Subsets and EDSS Scores (Spearman Correlation).

Variable	Correlation coefficient (r)	*P* value
CD4+ T cell percentage	–0.071	0.630
CD8+ T cell percentage	0.196	0.183
CD4+/CD8+ ratio	–0.345	0.016*
CD4+ absolute count	0.011	0.942
CD8+ absolute count	0.215	0.142
Disease duration (months)	–0.272	0.061

*P < 0.05, statistically significant. Note: Spearman's rank correlation coefficient was used for all analyses.

Individual T cell subset percentages showed weaker and non-significant associations with EDSS. CD8 + T cell percentage demonstrated a weak positive correlation approaching but not reaching statistical significance (*r* = 0.196, *p* = 0.183), while CD4 + T cell percentage showed minimal correlation (*r* = −0.071, *p* = 0.630). Absolute CD4 + and CD8 + T cell counts also showed no significant correlations with EDSS scores (*r* = 0.011, *p* = 0.942 and *r* = 0.215, *p* = 0.142, respectively). Disease duration showed a weak negative correlation with EDSS that did not reach statistical significance (*r* = −0.272, *p* = 0.061).

### 3.4 Multivariable regression analysis

Multivariable linear regression models were constructed to further evaluate the association between immunological variables and EDSS scores (Table 4). In the selected covariate-adjusted model including CD4+/CD8 + ratio, CD8 + T-cell percentage, and disease duration, CD4+/CD8 + ratio showed a negative association with EDSS scores, but this association did not reach statistical significance, *β* = −0.350, SE = 0.198, *p* = 0.084, 95% CI: −0.749 to 0.049. This negative coefficient indicates that lower CD4+/CD8 + ratio values tended to be associated with higher EDSS scores within the model. However, because the confidence interval crossed zero and treatment-related variables were not included in this primary model, this finding should be interpreted as an exploratory association rather than evidence of an independent treatment-adjusted relationship.

**Table 4:** Multivariable Linear Regression Analysis of Factors Associated With EDSS Score.

Variable	β	SE	*P* value	95% CI
Constant	3.248	0.887	0.001	1.461, 5.035
CD4+/CD8+ ratio	–0.350	0.198	0.084	–0.749, 0.049
CD8+ T cell percentage	0.041	0.028	0.146	–0.015, 0.098
Disease duration (months)	–0.021	0.014	0.142	–0.050, 0.007

Model R^2^ = 0.185, Adjusted R^2^ = 0.130. Abbreviations: β, unstandardized coefficient; CI, confidence interval; SE, standard error. This selected covariate-adjusted model did not include treatment exposure variables; DMT-related analyses are presented as exploratory supplementary sensitivity analyses.

CD8 + T cell percentage showed a trend toward positive association with EDSS (*β* = 0.041, SE = 0.028, *p* = 0.146, 95% CI: −0.015 to 0.098). Disease duration demonstrated a non-significant negative trend (*β* = −0.021, SE = 0.014, *p* = 0.142, 95% CI: −0.050 to 0.007). The overall model demonstrated modest explanatory power, with R^2^ = 0.185 and adjusted R^2^ = 0.130, indicating that the examined immunological variables accounted for a limited proportion of the variance in EDSS scores. These findings suggest a possible association between peripheral immune profile and disability status, but they do not establish CD4+/CD8 + ratio as a treatment-independent predictor of disability.

### 3.5 Stratified analysis by disability severity

**Figure 1:**
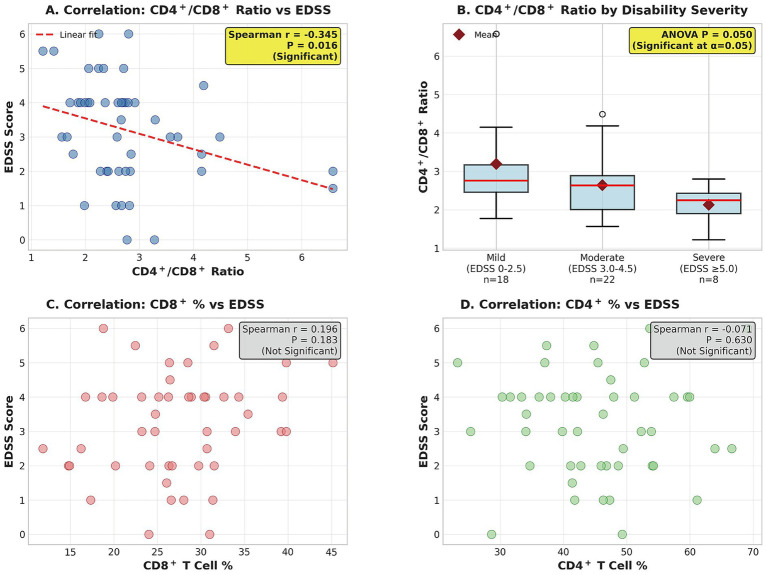
Correlation analysis between T cell subsets and clinical disability in MS patients. **(A)** Scatter plot showing the correlation between CD4+/CD8 + ratio and EDSS scores (*n* = 48). Spearman correlation revealed a significant negative correlation (*r* = −0.345, *p* = 0.016). The red dashed line indicates the fitted regression line. **(B)** Box plot comparing CD4+/CD8 + ratios across disability severity groups: mild (EDSS 0–2.5, *n* = 18), moderate (EDSS 3.0–4.5, *n* = 22), and severe (EDSS ≥5.0, *n* = 8). Red diamonds indicate group means. One-way ANOVA showed a borderline difference across groups (*p* = 0.050). **(C)** Scatter plot of CD8 + T cell percentage versus EDSS scores. No significant correlation (*r* = 0.196, *p* = 0.183). **(D)** Scatter plot of CD4 + T cell percentage versus EDSS scores. No significant correlation (*r* = −0.071, *p* = 0.630). The CD4+/CD8 + ratio, but not individual CD4 + or CD8 + percentages, significantly correlated with clinical disability in MS patients. Spearman correlation was used for panels **A**, **C** and **D**; one-way ANOVA was used for panel **B**. *p* < 0.05 was considered statistically significant.

To further elucidate the relationship between T cell subsets and clinical disability, patients were stratified into three groups based on EDSS scores: mild disability (EDSS 0–2.5, n = 18), moderate disability (EDSS 3.0–4.5, n = 22), and severe disability (EDSS ≥5.0, n = 8). Analysis of CD4+/CD8 + ratios across these groups revealed a progressive decline with increasing disability severity (Figure 1; Table 5).

**Table 5:** T Cell Subset Profiles Stratified by EDSS Disability Groups.

Disability Group	n	CD4+/CD8+ ratio	CD4+ (%)	CD8+ (%)
Mild (EDSS 0-2.5)	18	3.19 ± 1.37	48.00 ± 9.62	23.96 ± 6.47
Moderate (EDSS 3.0-4.5)	22	2.64 ± 0.81	42.94 ± 9.88	28.76 ± 6.60
Severe (EDSS ≥5.0)	8	2.13 ± 0.56	45.42 ± 13.69	30.71 ± 8.72
*P value (ANOVA)*	—	0.050*	0.324	0.037

*P = 0.050, indicating a borderline difference across groups. Values are presented as mean ± SD.

The mild disability group demonstrated a mean CD4+/CD8 + ratio of 3.19 ± 1.37, the moderate disability group showed 2.64 ± 0.81, and the severe disability group exhibited the lowest ratio of 2.13 ± 0.56. One-way ANOVA demonstrated a borderline difference across groups (*F* = 3.214, *p* = 0.050), indicating a trend toward lower CD4+/CD8 + ratios with increasing disability. CD8 + T-cell percentage also differed across EDSS severity groups, with values of 23.96 ± 6.47%, 28.76 ± 6.60%, and 30.71 ± 8.72% in the mild, moderate, and severe disability groups, respectively (*p* = 0.037). In contrast, CD4 + T-cell percentage did not differ significantly across the three groups (47.99 ± 9.62%, 42.94 ± 9.88%, and 45.42 ± 13.69%, respectively; *p* = 0.324).

These findings support a graded pattern of association between lower CD4+/CD8 + ratio and greater disability severity in this cohort. However, given the limited subgroup sample sizes, particularly in the severe disability group, and the absence of formal multiple-comparison correction, these stratified results should be interpreted cautiously. Representative brain MRI images for the mild, moderate, and severe EDSS groups are provided in Supplementary Figure S1 to illustrate the radiological context of the disability stratification used in the present analysis.

### 3.6 Treatment-related sensitivity analyses

Because DMT exposure may substantially influence peripheral lymphocyte subsets, exploratory treatment-related sensitivity analyses were performed (Supplementary Tables S2–S5). When current DMT status was added to the regression model, the association between CD4+/CD8 + ratio and EDSS was attenuated and remained non-significant (*β* = −0.214, SE = 0.202, *p* = 0.295). When exposure to strong lymphocyte-modifying DMTs, defined as fingolimod or ofatumumab use, was added to the model, the association between CD4+/CD8 + ratio and EDSS was further attenuated (*β* = −0.202, SE = 0.167, *p* = 0.233). In a model including DMT-type indicators, the association between CD4+/CD8 + ratio and EDSS also remained non-significant (*β* = −0.167, SE = 0.173, *p* = 0.341).

An additional sensitivity analysis was performed after excluding patients receiving fingolimod or ofatumumab. In this restricted cohort, the unadjusted Spearman correlation between CD4+/CD8 + ratio and EDSS was directionally consistent but no longer statistically significant (r = −0.306, *p* = 0.069). In the corresponding covariate-adjusted regression model, CD4+/CD8 + ratio was not significantly associated with EDSS (*β* = −0.185, SE = 0.196, *p* = 0.353).

Taken together, these sensitivity analyses indicate that the observed relationship between CD4+/CD8 + ratio and EDSS is sensitive to treatment-related adjustment and exclusion of patients receiving lymphocyte-modifying therapies. Therefore, residual confounding by DMT exposure cannot be excluded, and the association between CD4+/CD8 + ratio and EDSS should be interpreted as exploratory.

## 4 Discussion

This cross-sectional study investigated the relationship between peripheral blood T cell subset distributions and clinical disability in MS patients. The principal finding was an unadjusted statistically significant negative association between CD4+/CD8 + ratio and EDSS scores, indicating that lower CD4+/CD8 + ratios were associated with greater clinical disability in this cohort. However, this association was attenuated and no longer reached statistical significance in the selected covariate-adjusted multivariable model. In addition, stratified analysis showed a progressive decline in CD4+/CD8 + ratio across disability severity groups. Together, these findings suggest that CD4+/CD8 + ratio may represent an exploratory peripheral immune indicator associated with disability status in MS. However, because the study was cross-sectional, because treatment exposure was heterogeneous, and because the multivariable and sensitivity analyses did not confirm a treatment-independent association, these observations should be interpreted as exploratory associations rather than evidence of a causal relationship between peripheral immune status and cumulative neurological disability. The contribution of this study is to provide clinically contextualized evidence linking a simple peripheral T-cell ratio with standardized disability assessment, while clearly defining its interpretation as an exploratory peripheral immune indicator rather than a direct measure of CNS pathology or a treatment-independent biomarker.

**Figure 2:**
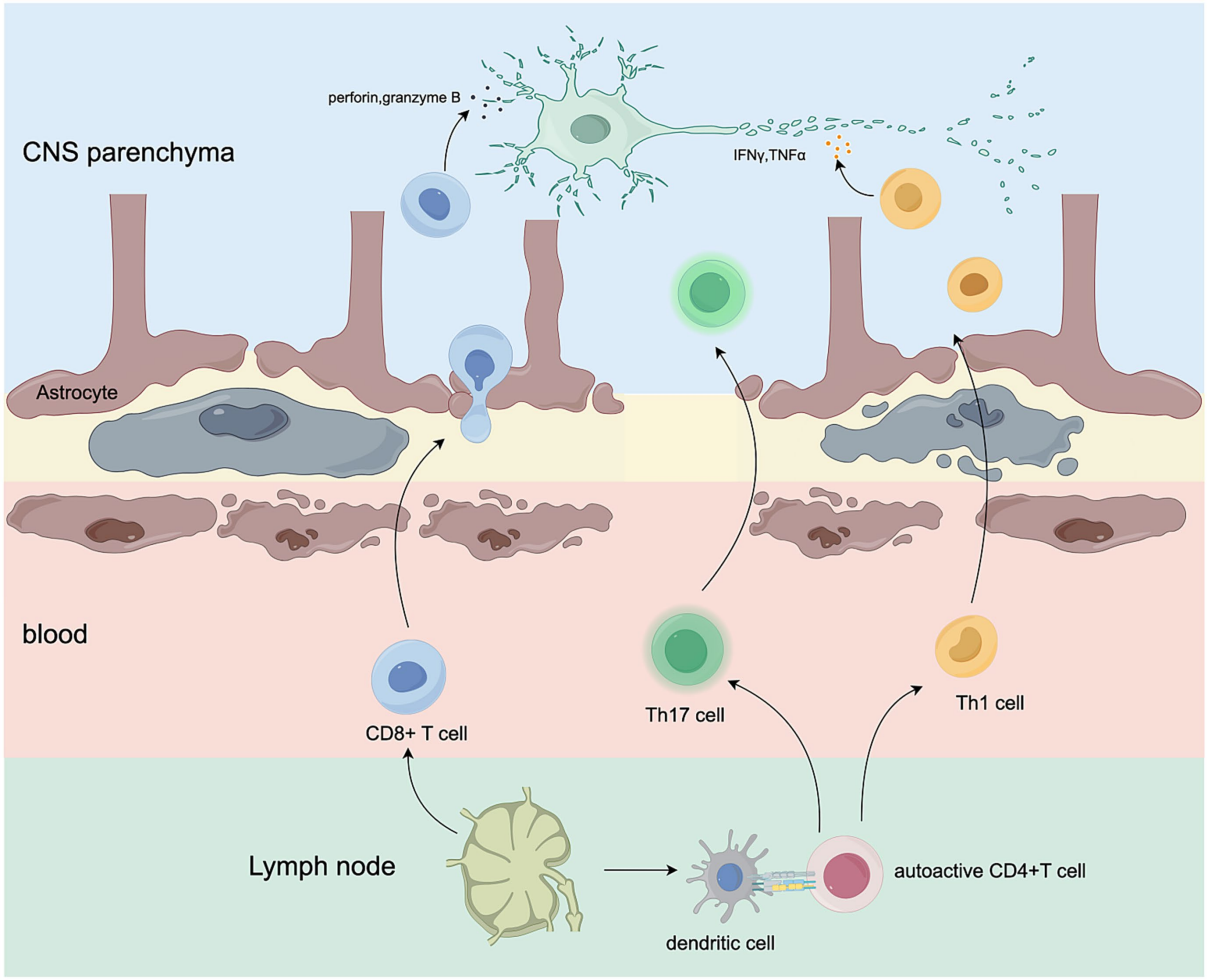
Schematic diagram illustrating the potential relationship between peripheral CD4+/CD8 + ratio and disability status in multiple sclerosis. This schematic summarizes the proposed relationship among peripheral immune imbalance, altered CD4+/CD8 + ratio, CNS-compartmentalized immune activity, neuroinflammation, and clinical disability in MS. Peripheral blood CD4+/CD8 + ratio is presented as a potential peripheral immunological indicator associated with disability status rather than a direct surrogate of pathogenic immune activity within the CNS. The diagram is intended to support interpretation of the observed cross-sectional association between CD4+/CD8 + ratio and EDSS scores.

The observed negative correlation between CD4+/CD8 + ratio and EDSS scores has potential biological and clinical implications. In MS pathogenesis, CD4 + T helper cells and CD8 + cytotoxic T cells play distinct but complementary pathogenic roles (3–5). CD4 + T cells, particularly Th1 and Th17 subsets, are crucial for orchestrating inflammatory responses and recruiting other immune cells to the CNS (5, 6). CD8 + T cells are abundant in MS lesions and can directly cause axonal damage and oligodendrocyte death through cytotoxic mechanisms (8, 9). The reduced CD4+/CD8 + ratio observed in patients with higher EDSS scores may reflect several pathophysiological processes. First, preferential expansion or persistence of CD8 + T cells could indicate ongoing cytotoxic immune activity contributing to neurodegeneration and disability accumulation. Second, relative CD4 + T cell depletion might reflect immune dysregulation, potentially including impaired regulatory T cell function, which normally helps control autoimmune inflammation (19, 20). Nevertheless, these biological interpretations should be considered hypothesis-generating, because DMT exposure may also alter CD4 + and CD8 + T-cell distributions and thereby influence the CD4+/CD8 + ratio independently of disability severity. At the same time, peripheral blood measurements capture only one compartment of the immune response. Most pathogenic CD4 + and CD8 + T cells involved in MS-related neuroinflammation are located within the CNS compartment rather than directly measured in the circulation. Therefore, the peripheral CD4+/CD8 + ratio should not be interpreted as a direct surrogate of intracerebral immune pathology, but rather as a peripheral immune indicator that may be associated with broader disease-related immune status. To clarify this interpretation, a schematic diagram has been added to illustrate the hypothesized relationship among peripheral immune imbalance, altered CD4+/CD8 + ratio, CNS-compartmentalized inflammation, and disability status in MS (Figure 2).

Our findings align with some previous investigations suggesting altered T cell homeostasis in MS patients with more severe disease (10, 11). However, results in the literature have been inconsistent, with some studies reporting no clear associations between peripheral T cell subsets and disability (14, 15). Several factors may explain these discrepancies. First, cross-sectional designs capture only a single time point and may not fully represent the dynamic nature of T cell populations during different disease phases. Second, treatment effects significantly confound peripheral immune profiles, as approximately 73% of the patients were receiving DMTs. Different therapeutic agents exert distinct effects on lymphocyte distributions. Fingolimod has been shown to alter lymphocyte subset composition through sequestration of lymphocytes in lymphoid tissues, and pretreatment T-cell subset composition may also influence subsequent treatment responsiveness. Anti-CD20-directed therapies may substantially modify circulating immune-cell profiles, and dimethyl fumarate has also been reported to affect peripheral lymphocyte subpopulations in relapsing MS. These findings from previous studies support the concern that DMT exposure may have contributed to the peripheral immune patterns observed in the present cohort and may have influenced the association between CD4+/CD8 + ratio and EDSS (21–26). Third, disease heterogeneity is substantial, with different MS subtypes potentially involving distinct immunopathological mechanisms (27). In the present cohort, patients received teriflunomide, fingolimod, or ofatumumab, and the heterogeneous immunological effects of these agents should be taken into account when interpreting the findings. This issue is particularly relevant because fingolimod and anti-CD20 therapy may have stronger and more direct effects on circulating lymphocyte subsets than some other DMTs. Accordingly, we performed exploratory sensitivity analyses incorporating current DMT status, strong lymphocyte-modifying DMT exposure, and DMT type, as well as an additional analysis excluding patients receiving fingolimod or ofatumumab. These analyses showed that the association between CD4+/CD8 + ratio and EDSS was attenuated after accounting for treatment-related variables, supporting the interpretation that residual treatment-related confounding cannot be excluded.

The stratified analysis demonstrating progressive CD4+/CD8 + ratio decline across disability groups is particularly informative. The graded decline across mild, moderate, and severe disability groups supports a possible relationship between lower CD4+/CD8 + ratio and greater disability burden in this cohort. Patients with mild disability showed the highest mean CD4+/CD8 + ratio, whereas those with severe disability showed the lowest mean value. In addition, CD8 + T-cell percentage differed across EDSS severity groups, whereas CD4 + T-cell percentage did not show a significant between-group difference. This pattern may reflect complex immune changes across different stages of disease, but the interpretation remains tentative because no matched healthy control group was included, subgroup sample sizes were limited, and no formal multiple-comparison correction was applied.

Importantly, individual CD4 + and CD8 + T cell percentages showed weaker associations with EDSS than the CD4+/CD8 + ratio in unadjusted analyses, highlighting that the ratio, rather than absolute subset proportions, may capture additional clinically relevant information. This suggests that the balance between CD4 + and CD8 + T cells may be more informative than the absolute levels of either subset alone. The CD4+/CD8 + ratio integrates information about both helper and cytotoxic T cell compartments and may better reflect overall immune dysregulation relevant to disease severity. Because no age- and sex-matched healthy control group was included, the present findings should be interpreted within the study cohort rather than against assumed reference values from healthy populations. Longitudinal studies will be necessary to determine whether the observed CD4+/CD8 + ratio pattern reflects dynamic immune changes across disease progression or treatment exposure. Moreover, because the CD4+/CD8 + ratio in the present study was calculated from absolute CD4 + and CD8 + T-cell counts, future studies should standardize whether this ratio is derived from absolute counts or flow-cytometric percentages to improve comparability across studies.

Several limitations warrant acknowledgment. The cross-sectional design precludes establishing temporal relationships or causality. EDSS reflects cumulative neurological impairment and functional reserve, whereas peripheral immune-cell distributions may vary dynamically over time and may be influenced by recent biological or therapeutic factors. Therefore, a single peripheral blood measurement cannot fully capture the complexity of long-term disability accumulation in MS. Longitudinal studies examining whether baseline CD4+/CD8 + ratios predict subsequent disability progression would provide more definitive evidence of clinical utility (28). The moderate sample size (n = 48) limited statistical power for subgroup analyses, particularly for progressive MS subtypes. Treatment heterogeneity represents an important source of potential confounding, as different DMTs have distinct effects on peripheral lymphocyte populations, and some therapies may exert persistent immunological effects beyond active treatment exposure. Although a multivariable regression model was performed, treatment exposure was not fully adjusted for in the final model because DMT status, DMT type, and treatment duration were not included as covariates. This limitation was mainly related to the limited sample size and heterogeneous treatment exposure in the present cohort. Exploratory treatment-related sensitivity analyses were performed, but these analyses were limited by small subgroup sizes and should not be considered definitive adjustment for treatment exposure. After incorporating treatment-related variables or excluding patients receiving fingolimod or ofatumumab, the association between CD4+/CD8 + ratio and EDSS was attenuated and remained non-significant. Therefore, residual confounding by DMT exposure cannot be excluded. Studies in treatment-naïve cohorts or with stratification by specific DMT types would provide clearer insights. In particular, a fully treatment-naïve cohort or a cohort uniformly exposed to a single defined DMT over a standardized duration would be methodologically more appropriate for determining whether CD4+/CD8 + ratio is independently associated with disability status. Because of the limited sample size and subgroup imbalance, additional analyses stratified by EDSS subgroup, MS subtype, or treatment category should still be considered exploratory, and this should be considered when interpreting the robustness of the findings. We assessed only basic T cell subsets without detailed phenotypic characterization of regulatory T cells, memory subsets, or effector populations, which might reveal more specific associations with disability (29). In addition, disability was assessed using EDSS alone. Although EDSS is widely used in MS research and clinical practice, it has recognized limitations, including limited sensitivity to non-ambulatory disability domains, non-linearity, and inter-rater variability. Future studies may benefit from incorporating multidimensional disability measures such as EDSS-Plus to provide a more comprehensive assessment of neurological impairment. The lack of comprehensive MRI data, including volumetric analysis and lesion location mapping, also limited the ability to correlate immunological findings with structural CNS damage patterns. Finally, the cohort comprised predominantly Asian patients from a single center, which may limit generalizability to other populations (30).

Despite these limitations, the present findings have potential clinical implications. If validated in larger longitudinal studies, CD4+/CD8 + ratio measurement may serve as a readily accessible exploratory peripheral immune marker for disability assessment in MS. Unlike MRI, which requires specialized equipment and expertise, flow cytometric T cell profiling can be performed in most clinical laboratories with relatively low cost and rapid turnaround. CD4+/CD8 + ratio monitoring might complement EDSS assessment by providing an objective immunological correlate of disability status. However, the present data do not support using CD4+/CD8 + ratio as a stand-alone or treatment-independent biomarker in routine clinical practice. This practical accessibility represents a clinically relevant aspect of the study, particularly for exploratory biomarker research in settings where advanced imaging or cerebrospinal fluid biomarkers may not be routinely available. Serial CD4+/CD8 + ratio measurements may also be of interest for future studies evaluating disease progression or treatment response, although this possibility requires prospective validation with careful control of DMT exposure, treatment duration, prior treatment history, and washout status.

Future research directions should include: (1) longitudinal cohort studies examining whether CD4+/CD8 + ratio predicts future disability accumulation and disease progression; (2) detailed phenotypic characterization of T cell subsets, including regulatory T cells, memory subsets, and exhaustion markers; (3) investigation of treatment effects on CD4+/CD8 + ratio and whether ratio changes correlate with treatment responses; (4) integration of T cell profiling with other biomarkers (neurofilament light chain, GFAP, MRI measures) to develop comprehensive multimodal prognostic models; (5) comparison of peripheral blood findings with cerebrospinal fluid immune profiles to understand compartmentalization; and (6) validation in independent, ethnically diverse cohorts to establish generalizability (31–33). Future studies should also prioritize treatment-naïve populations or cohorts with uniform exposure to a single DMT and standardized treatment duration, because such designs would better separate disability-related immune alterations from treatment-related lymphocyte changes.

## 5 Conclusion

This study showed that peripheral blood CD4+/CD8 + ratio was negatively associated with clinical disability in this cross-sectional MS cohort, with lower ratios corresponding to higher EDSS scores. Stratified analysis further demonstrated a progressive decline in CD4+/CD8 + ratio across disability severity groups. Exploratory treatment-related sensitivity analyses also suggested that this association may be influenced by DMT exposure. These findings suggest that CD4+/CD8 + ratio may represent a potential exploratory peripheral immune indicator, associated with disability status in MS and may complement clinical evaluation and neuroimaging in future research settings. Longitudinal validation studies are warranted to further define the clinical relevance of CD4+/CD8 + ratio monitoring in relation to disease progression and treatment response, particularly in treatment-naïve cohorts or cohorts with standardized DMT exposure and treatment duration.

## Data Availability

The original contributions presented in the study are included in the article/Supplementary material, further inquiries can be directed to the corresponding author.

## Supplementary material

SUPPLEMENTARY FIGURE S1 Representative brain MRI images across EDSS disability severity groups in multiple sclerosis. **(A)** Representative brain MRI from a patient in the mild disability group (EDSS 0–2.5). **(B)** Representative brain MRI from a patient in the moderate disability group (EDSS 3.0–4.5). **(C)** Representative brain MRI from a patient in the severe disability group (EDSS ≥5.0).
