# Supplementary figures and images for: Association between peripheral blood T cell subsets and clinical disability in multiple sclerosis patients
Source: Front Neurol. 2026 Jul 20;17:1843351. doi: 10.3389/fneur.2026.1843351 (PMC13430141; doi:10.3389/fneur.2026.1843351)

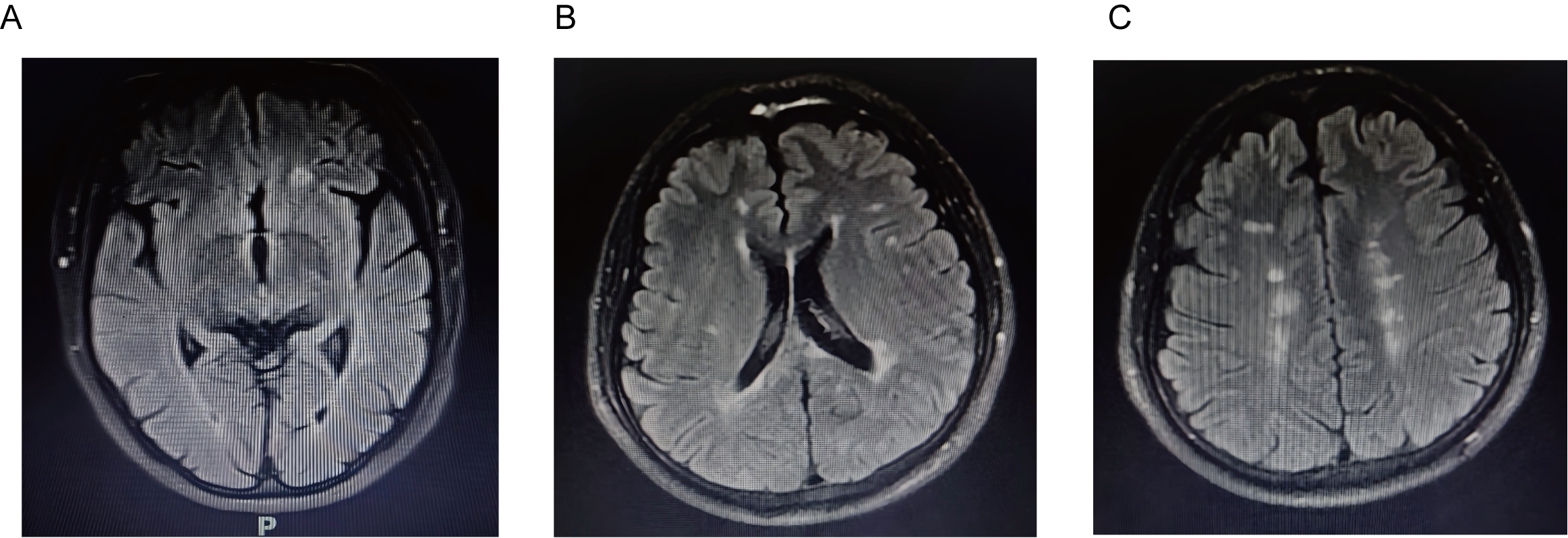

Supplement: SUPPLEMENTARY FIGURE S1 — Representative brain MRI images across EDSS disability severity groups in multiple sclerosis. (A) Representative brain MRI from a patient in the mild disability group (EDSS 0–2.5). (B) Representative brain MRI from a patient in the moderate disability group (EDSS 3.0–4.5). (C) Representative brain MRI from a patient in the severe disability group (EDSS ≥5.0). [file Image_1.TIF]
