# Supplementary material for: Association between peripheral blood T cell subsets and clinical disability in multiple sclerosis patients
Source: Front Neurol. 2026 Jul 20;17:1843351. doi: 10.3389/fneur.2026.1843351 (PMC13430141; doi:10.3389/fneur.2026.1843351)
Supplement: Supplementary file 2 [file Table_1.DOCX]

Supplementary Table S1. Current DMT exposure and treatment duration

| Current treatment group | n (%) | Treatment duration, months, mean ± SD | Range, months | Interpretation |
| --- | --- | --- | --- | --- |
| Untreated | 13 (27.1) | Not applicable | Not applicable | No current DMT agent recorded at enrollment |
| Teriflunomide | 23 (47.9) | 18.65 ± 6.17 | 6-28 | Current teriflunomide exposure |
| Fingolimod | 7 (14.6) | 12.57 ± 5.00 | 6-19 | Current fingolimod exposure |
| Ofatumumab | 5 (10.4) | 9.40 ± 2.70 | 6-13 | Current anti-CD20 exposure |
| Any current DMT | 35 (72.9) | — | — | Teriflunomide, fingolimod, or ofatumumab |

Abbreviations: DMT, disease-modifying therapy; SD, standard deviation.
