# Supplementary material for: Association between peripheral blood T cell subsets and clinical disability in multiple sclerosis patients
Source: Front Neurol. 2026 Jul 20;17:1843351. doi: 10.3389/fneur.2026.1843351 (PMC13430141; doi:10.3389/fneur.2026.1843351)
Supplement: Supplementary file 3 [file Table_2.DOCX]

Supplementary Table S2. Peripheral immune measures according to current DMT group

| Variable | Untreated (n=13) | Teriflunomide (n=23) | Fingolimod (n=7) | Ofatumumab (n=5) | P value (ANOVA) |
| --- | --- | --- | --- | --- | --- |
| EDSS score | 2.42 ± 1.59 | 2.89 ± 1.22 | 4.36 ± 0.85 | 5.00 ± 0.79 | <0.001 |
| CD4+/CD8+ ratio | 3.26 ± 1.38 | 2.64 ± 0.98 | 2.30 ± 0.53 | 2.68 ± 1.01 | 0.228 |
| CD4+ T-cell percentage | 46.95 ± 9.01 | 44.66 ± 11.03 | 41.03 ± 10.99 | 49.45 ± 11.79 | 0.518 |
| CD8+ T-cell percentage | 26.87 ± 7.37 | 26.75 ± 7.58 | 29.05 ± 6.49 | 28.36 ± 8.62 | 0.884 |
| CD4+ absolute count, cells/μL | 745.66 ± 213.62 | 767.71 ± 206.02 | 765.97 ± 255.21 | 759.78 ± 184.64 | 0.992 |
| CD8+ absolute count, cells/μL | 274.85 ± 155.26 | 317.52 ± 103.14 | 339.29 ± 106.70 | 332.20 ± 174.18 | 0.663 |
| Lymphocyte measure in dataset | 26.53 ± 7.53 | 23.90 ± 7.80 | 25.89 ± 5.27 | 26.70 ± 5.94 | 0.696 |

Note: Because of small subgroup sizes and treatment indication bias, these comparisons are descriptive and exploratory. EDSS differed across DMT groups, indicating potential clinical confounding by treatment selection.
