# Supplementary material for: Association between peripheral blood T cell subsets and clinical disability in multiple sclerosis patients
Source: Front Neurol. 2026 Jul 20;17:1843351. doi: 10.3389/fneur.2026.1843351 (PMC13430141; doi:10.3389/fneur.2026.1843351)
Supplement: Supplementary file 4 [file Table_3.DOCX]

Supplementary Table S3. Regression sensitivity analyses incorporating treatment exposure

| Model | Variables added to selected model | R^2^ | Adjusted R² | CD4+/CD8+ β | CD4+/CD8+ P value | Treatment variable β | Treatment variable P value |
| --- | --- | --- | --- | --- | --- | --- | --- |
| A | CD4+/CD8+ ratio + CD8% + disease duration | 0.185 | 0.130 | -0.350 | 0.084 | Not included | Not included |
| B | Model A + current DMT status | 0.260 | 0.191 | -0.214 | 0.295 | Current DMT: 0.967 | 0.043 |
| C | Model A + strong lymphocyte-modifying DMT (fingolimod/ofatumumab) | 0.455 | 0.405 | -0.202 | 0.233 | Strong DMT: 1.829 | <0.001 |
| D | Model A + DMT type indicators vs untreated | 0.488 | 0.414 | -0.167 | 0.341 | Fingolimod: 1.781; Ofatumumab: 2.607 | 0.003; <0.001 |

Note: The selected model included CD4+/CD8+ ratio, CD8+ T-cell percentage, and disease duration. Treatment-adjusted models were exploratory because of the small sample size and heterogeneous treatment exposure.
